# Orbital inflammatory disease secondary to epidemic keratoconjunctivitis in an adult patient: case report

**DOI:** 10.11604/pamj.2021.38.166.27121

**Published:** 2021-02-12

**Authors:** Temiloluwa Moyosoreoluwa Abikoye

**Affiliations:** 1 Metro Eye Centre, Lagos, Nigeria,; 2 Guinness Eye Centre, Lagos University Teaching Hospital, Lagos, Nigeria

**Keywords:** Orbital inflammation, molecular mimicry, conjunctivitis, epidemic keratoconjunctivitis, case report

## Abstract

Orbital inflammatory disease, sequel to epidemic keratoconjunctivitis is an uncommon finding in adult patients. A 36-year-old male presented at the clinic with a 4-day history of left ocular pain and a one-month history of left eye redness and watering. Visual acuity in the eye was 6/5, with reduced red-color saturation and light brightness appreciation. Left eye examination showed periorbital fullness, a palpably enlarged and tender lacrimal gland, conjunctival follicles with pseudomembranes, and restriction of extraocular motility. Magnetic resonance imaging showed homogenous enhancement of the left lacrimal gland, lateral rectus muscle, pre and post-septal soft tissues. A diagnosis of left orbital inflammatory disease secondary to epidemic keratoconjunctivitis was made and patient was treated with high dose oral steroids over the course of 7 weeks, with complete resolution of clinical symptoms. In conclusion, orbital inflammatory disease can develop following epidemic keratoconjuctivitis in adults with good clinical response to oral steroids. Clinicians should have a high index of suspicion when assessing adult patients for orbital inflammatory disease.

## Introduction

Orbital Inflammatory Disease (OID) is a clinical syndrome characterized by inflammation of the extraocular orbit and adnexa [1,2]. While literature abound on the idiopathic and systemic etiologies, infectious agents are less common causes and should be considered in all cases of orbital inflammation [2-4]. Viral infections have been implicated, through the mechanism of molecular mimicry, as causes of OID [5]. However, while adenoviral conjunctivitis has been reported to cause orbital inflammation in children [6], to the author´s knowledge, only one case of an adult developing OID secondary to epidemic keratoconjunctivitis (EKC) has been reported in literature [7]. Herein, the author reports a case of OID secondary to epidemic keratoconjunctivitis in an adult patient.

## Patient and observation

**Figure 1:**
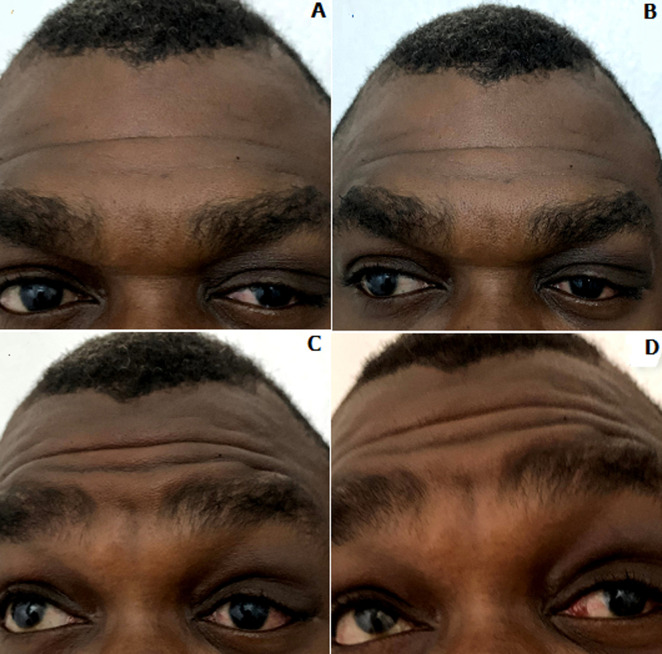
S-shaped ptosis and restriction of left extraocular motility; A) in primary gaze; B) in right gaz; C) in right elevation; D) in left elevation

A 36-year-old male presented with a four-day history of left eye pain and blurring of vision. Pain was of gradual onset, exacerbated by eye movement and bright light. He also had a preceding one-month history of redness and watering of the same eye. There was no recent history of respiratory or febrile illness. He was previously seen by an optometrist and referred due to the acute worsening of his symptoms. Patient is a known hypertensive on medication. Visual acuity was 6/5 in both eyes, with subjective blurring of left eye vision, decreased red-color desaturation and light brightness appreciation (6/10 and 7/10, respectively). Examination of the left eye revealed periorbital fullness, a palpably enlarged, tender lacrimal gland, S-shaped ptosis and restriction of extraocular motility (Figure 1). Slit lamp examination showed mild conjunctival hyperemia with inferior fornix pseudomembranes, superior and inferior tarsal follicles, and fine, staining, epithelial keratitis. There was no proptosis. Examination of the right eye did not reveal any remarkable findings.

**Figure 2:**
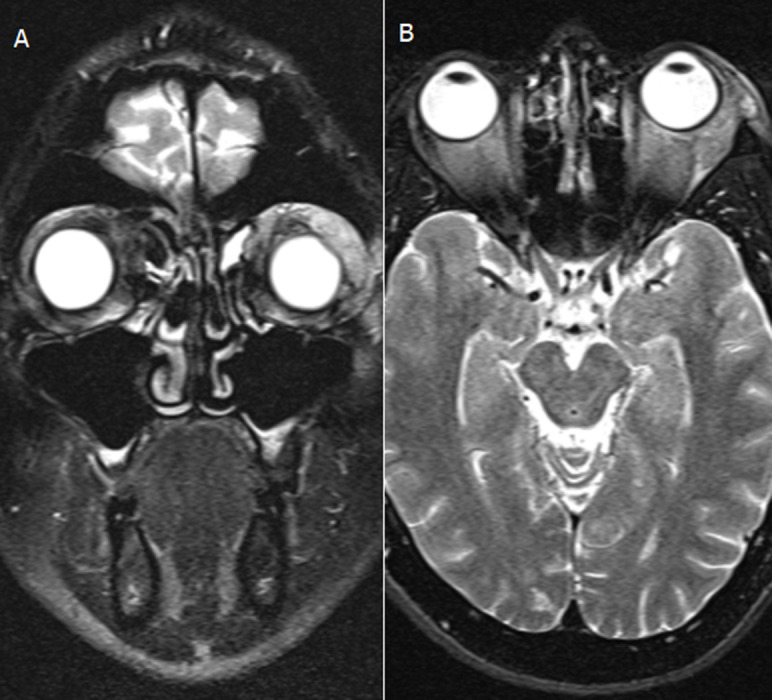
T2W magnetic resonance imaging of the brain and orbit; A) coronal cut showing enhancement and enlargement of the left lacrimal gland; B) axial cut showing enhancement and enlargement of the left lateral rectus and surrounding soft tissue

Central visual field tests showed enlargement of the blind spot in the left eye. Contrast magnetic resonance imaging of the left orbit showed enhancement of the lacrimal gland; lateral rectus; pre- and post-septal soft tissue in the superolateral and medial quadrants (Figure 2). The complete blood count, electrolyte sedimentation rate, retroviral screening and random blood sugar tests were all within normal limits.

A diagnosis of left orbital inflammatory disease secondary to EKC was made. The patient was commenced on high dose oral prednisolone of 100mg daily with a weekly taper over 7 weeks. Regular clinic follow appointments were scheduled. The patient´s presenting complaints had fully resolved by the two-week follow up appointment. Therapy was well tolerated- blood pressure assessment and random blood sugar tests were normal. Visual acuity was 6/5 in both eyes, with equal light brightness appreciation and red-color desaturation bilaterally. Ocular examination revealed a residual limitation of adduction, but otherwise normal ocular findings. Patient presented for a final visit after completion of steroid therapy, 7 weeks after institution. Ocular symptoms and signs were all resolved and intraocular pressure remained normal. He was subsequently discharged from follow up.

## Discussion

EKC is the most frequent reason for patient presentation at the ophthalmic emergency room, globally [8]. The chief complaints are eyelid swelling, eye redness, watering and itching, while the most prevalent clinical signs are conjunctival hyperemia, follicles and pseudomembranes [7,8]. Tests are frequently carried out for the evaluation of the disease, particularly in developed countries, however diagnosis is mainly clinical, based on history and examination findings [8,9]. The disease is usually self-limiting and only a few cases of a secondary inflammatory sequelae have been reported in literature [6,7,10]. OID, by comparison, is a less common disease entity, accounting for up to 6% of all orbital diseases [4]. The clinical spectrum includes dacryoadenitis, myositis, scleritis, optic neuritis and diffuse orbital inflammation [2,4]. While pain and diplopia are the commonest reasons for presentation, periorbital swelling is the most frequently observed clinical sign, followed by proptosis and restriction of extraocular motility [2].

Reports of OID following EKC are rare, particularly in adults [7]. The patient presented had a one-month history of symptoms consistent with viral conjunctivitis, prior to the onset of pain and blurring, which necessitated his referral. Interesting in the case was that in spite of the presence of extraocular motility restriction, diplopia was not a presenting complaint (rather, elicited during the examination). Also, while the patient had diffuse orbital inflammation and optic nerve involvement, the `striking´ signs of OID- proptosis and chemosis [5] were absent. Only 38% of patients present with the complaint of diplopia, while chemosis is present in less than a third of cases [2]. A high index of suspicion, as well as full clinical examination, is important in all cases of periorbital fullness.

Systemic corticosteroid therapy is the primary treatment for OID, by oral or intravenous routes of administration, usually over the course of 6-12 weeks [3,4,7]. While the treatment of refractory cases of OID with immune modulatory agents has been documented, [3,4] there is a paucity of literature on OID secondary to EKC, and so no reports of treatment-failure with steroid therapy. The patient above recovered fully, without rebound inflammation, during the steroid taper. An important differential of OID is orbital cellulitis, a potentially life-threatening disease [4]. As both orbital diseases have similar clinical and radiological signs, this distinction is important to make. Steroid monotherapy in the setting of orbital cellulitis could have catastrophic consequences. Indeed, antibiotic therapy has been instituted initially, in cases that the non-infective aetiology of the orbital disease was uncertain [1,6,7]. It is certainly safer to do so. The absence of fever and leukocytosis, [4] as well as the `non-toxic´ general appearance of the patient, were convincing indicators that this was not a case of orbital cellulitis. The subsequent response to steroid therapy was the ultimate proof.

While investigations like polymerase chain reaction (PCR) and rapid adenovirus antigen or nucleic acid detection tests were not carried out, the clinical diagnosis of viral conjunctivitis is widespread among ophthalmologists and accepted [8,9]. The patient´s symptoms and signs were consistent with EKC. A strong positive correlation has been reported between the presence of conjunctival pseudomembranes and a positive adenovirus result on PCR testing.

## Conclusion

Orbital inflammatory disease can occur sequelae to epidemic viral conjunctivitis in adult patients. There is a good response to oral steroids, with complete resolution of clinical features. Ophthalmologists should have a high index of suspicion when evaluating patients presenting with orbital inflammation.
